# Genome-wide identification and analysis of the *SUPPRESSOR of MAX2 1-LIKE* gene family and its interaction with DWARF14 in poplar

**DOI:** 10.1186/s12870-023-04118-w

**Published:** 2023-02-22

**Authors:** Maotong Sun, Dongyue Wang, Cuishuang Liu, Yuan Liu, Muge Niu, Jinnan Wang, Jihong Li

**Affiliations:** 1 College of Forestry, Shandong Agricultural University, Taian, 271018 Shangdong China; 2 Mountain Tai Forest Ecosystem Research Station of State Forestry and Grassland Administration, Shandong Agricultural University, Taian, 271018 Shangdong China; 3 State Forestry and Grassland Administration Key Laboratory of Silviculture in Downstream Areas of the Yellow River, Taian, 271018 Shangdong China

**Keywords:** *SMXL* gene family, Strigolactones, Poplar, Expression pattern, Protein interaction

## Abstract

**Background:**

Strigolactones (SLs) are important phytohormones that can regulate branch development in plants. Although SUPPRESSOR of MAX2 1-LIKE proteins (SMXLs) play a crucial role in SL signaling transduction, the *SMXL* gene family has not been well characterized in poplar.

**Results:**

In this study, 12 members of the poplar *SMXL* gene family were identified and phylogenetically classified into four clades. Motif and 3D structural analyses revealed that PtSMXL proteins are structurally very conserved; however, the P-loop NTPase domain at the C-terminal was found to vary substantially among clades. A genomic collinearity analysis indicated that *PtSMXL* gene family members have expanded during recent genome doubling events in poplar, with all gene pairs subsequently undergoing purifying selection. According to a *Cis*-element analysis, *PtSMXL* promoters contain many light-responsive elements. In an expression pattern analysis, all 12 *PtSMXL* genes displayed tissue-specific expression, especially *PtSMXL8a*. *PtSMXL7b* expression was significantly downregulated after axillary bud growth begins. In addition, the expressions of *PtSMXL7b* and *PtSMXL8a* were highly induced by 2 μM GR24, a synthetic SL analog, thus suggesting that these genes are involved in SL-regulated axillary bud growth. In a yeast two-hybrid assay, only PtSMXL7b in clade II was able to interact with the SL receptor PtD14a in an SL dependent manner, which indicates that *PtSMXL7b* may be the functional homolog of *D53/SMXL6/7/8* in poplar. Finally, we established its ability to affect axillary bud growth by constructing poplar overexpressing the *PtSMXL7b* gene.

**Conclusions:**

Our findings may inform future research on the functions of *SMXLs* in poplar, especially with respect to branch development.

**Supplementary Information:**

The online version contains supplementary material available at 10.1186/s12870-023-04118-w.

## Background

Tree crown shape and photosynthetic area are profoundly influenced by branch development [1]. Among the many factors that control branch development in plants, hormones play an important role [2]. Strigolactones (SLs) are carotenoid-derived terpenoid lactones that function as phytohormones to regulate a variety of physiological activities, including shoot branching [3]. Current studies have shown that SL signal transduction depends on interactions among several proteins, including SUPPRESSOR of MAX2 1-LIKE (SMXL), the key repressor in SL signaling [4, 5]. *SMXLs* constitute a conserved gene family widely present in land plants [6]. SMXL proteins contain well-conserved structures, including double Clp-N and P-loop NTPase domains [5, 7]. In addition, two conserved functional motifs have been identified in SMXLs: ETHYLENE-RESPONSE FACTOR Amphiphilic Repression (EAR) and RGKT (Arg-Gly-Lys-Thr) motifs [4, 8, 9]. These two motifs are indispensable for SMXL function. Deletion of either motif affects the number and angle of branches to branches to varying degrees in *Arabidopsis*, and the EAR motif is also an important identifying feature of SMXLs [6, 8].

Evolutionary studies have shown that the *SMXL* gene family originated from the ancient *SMAX1* gene of liverworts and mosses and then diversified in green plants, ultimately forming four characteristic clades in angiosperms [6]. Owing to functional differentiation, the four clades of *SMXLs* have different biological functions [7]. One of these clades, clade II containing *AtSMXL6/7/8* of *Arabidopsis* and *OsD53* of rice, is mainly involved in SL signaling [9]. When the SL receptor D14 senses SLs, it can interact with SMXLs and then SMXLs undergo degradation by ubiquitination, which activates SL downstream signaling [10, 11]. Mutation of *AtSMXL6/7/8*, repressors of SL signaling, leads to a reduced number of branches in *Arabidopsis*, whereas overexpression of stable *AtSMXL6* increases branching [8, 12]. In addition to regulating plant shoot branch number, the SL signal transduction pathway influenced by *SMXL* clade II has roles in the regulation of branching angle, lateral root growth, and leaf senescence [13–15]. *SMXL* clade I participates in the KARRIKIN (KAR) signaling pathway. KARs, which are small molecules that shares several features with SLs, all containing an essential butanolide ring. KARs are found in smoke derived from plant combustion and are able to promote seed germination [15, 16]. Members of clade I are degraded by ubiquitination in a manner similar to members of clade II; they regulate physiological activities, such as seed germination and seedling development [17, 18]. At present, the few reported studies on the functions of clade III and IV have been focused on their regulation of starch and anthocyanins accumulation and primary phloem formation [19, 20].

The number of *SMXL* family members varies extensively in plants, with 1 member present in the liverwort *Marchantia polymorpha* [21], 8 in *Arabidopsis* [22], and 10 in apple [23]. poplar, which has a fast growth rate and a straight trunk, is an excellent afforestation tree for temperate areas [24]. For poplar, branch number and angle are important factors related to planting density and crown shape [1], but little is known about the *SMXL* gene family and its effect on branching. In this study, we identified *SMXL* gene family members in poplar by bioinformatics methods. We then performed conserved structural domain, motif, phylogenetic, and expression analyses and validated yeast two-hybrid interactions between SL receptor proteins PtD14a/b and four members of clade II related to SL signaling. In addition to that, we also studied its function in axillary bud growth by using transgenic plants. The results of our study should extend understanding of *SMXLs* and provide a reference for breeding efforts related to branching traits in poplar.

## Results

### Identification and phylogenetic analysis of *PtSMXL* genes

To identify *SMXL* genes in poplar, an HMM model based on SMXL protein sequences of *Arabidopsis* was constructed and used as a query in a search against the *P. trichocarpa* genome. We then confirmed the presence of conserved double Clp-N and P-loop NTPase domains and the conserved EAR motif. We identified 12 *PtSMXL* genes in the *Populus* genome (Table 1). Predicted proteins encoded by these genes had amino acid lengths of 856 to 1141 residues, pI values varying from 6.18 to 8.64, and MWs ranging from 95.17 to 125.89 kDa. On the basis of instability Index II and GRAVY values, all *PtSMXL* genes encoded unstable hydrophilic proteins [25, 26].

**Table 1:** Physical and chemical characteristics of the 12 *PtSMXLs*

Gene	Gene ID	Protein length	Mw (KDa)	pI	Location	GRAVY	Instability index II
*PtSMXL1a*	Potri.018G097300	1049	114.91108	8.04	Chr18	−0.368	44.92
*PtSMXL1b*	Potri.006G175200	1049	114.94115	8.64	Chr06	−0.366	46.04
*PtSMXL3a*	Potri.006G204500	866	95.50497	6.58	Chr06	−0.346	60.91
*PtSMXL3b*	Potri.016G071800	861	95.61485	6.35	Chr16	−0.431	56.31
*PtSMXL3c*	Potri.008G017600	857	95.17066	7.25	Chr08	−0.431	55.26
*PtSMXL3d*	Potri.010G241600	856	95.51714	6.48	Chr10	−0.412	54.68
*PtSMXL4a*	Potri.018G140900	1021	114.27215	7.53	Chr18	−0.485	43.21
*PtSMXL4b*	Potri.006G073800	1017	114.11605	7.78	Chr06	−0.5	47.03
*PtSMXL7a*	Potri.009G046700	1115	121.94392	6.45	Chr09	−0.313	46.17
*PtSMXL7b*	Potri.001G252500	1117	122.48838	6.22	Chr01	−0.31	43.59
*PtSMXL8a*	Potri.008G069100	1141	125.89642	6.8	Chr08	−0.345	50.05
*PtSMXL8b*	Potri.010G188200	1136	125.3809	6.18	Chr10	−0.326	48.16

**Fig. 1:**
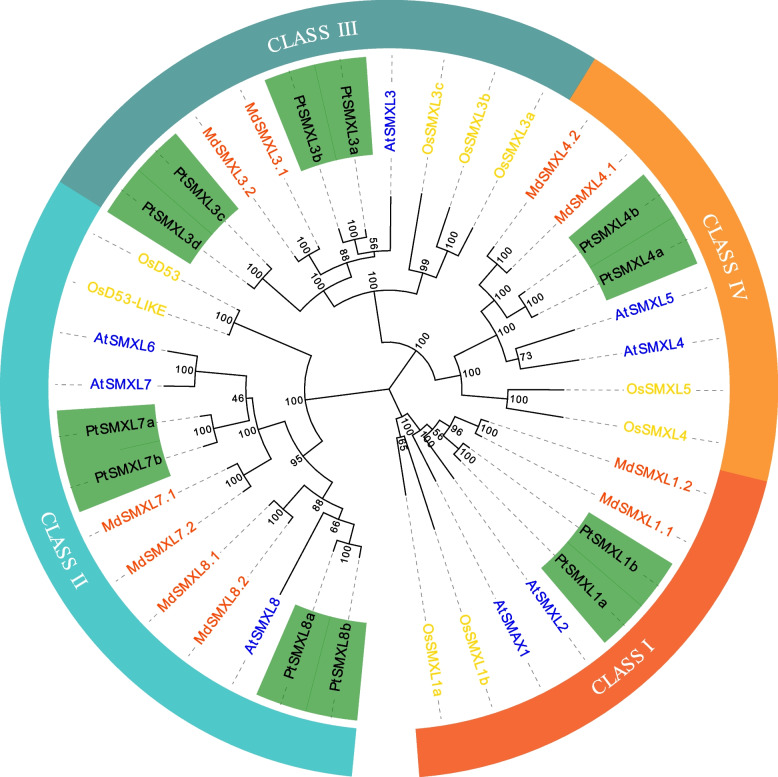
Phylogenetic tree analysis of 39 SMXL proteins from poplar, *Arabidopsis*, rice, and apple. SMXL proteins from different species were marked by different colors, poplar (green), *Arabidopsis* (blue), rice (yellow), apple (red). Different clades were highlighted in different colors

To classify *PtSMXL* genes and investigate their phylogenetic relationships, we constructed a phylogenetic tree based on 39 aligned protein sequences from *Arabidopsis*, rice, apple, and poplar. Consistent with previous reports, the 39 SMXL proteins from the four species were clustered into four clades (Fig. 1) [6]. Two members of clade I from poplar were most closely related to those of apple. In clade II, four members from poplar were divided into two subclades, thus indicating that *PtSMXL7a/b* and *PtSMXL8a/b* were closest to *AtSMXL6/7* and *AtSMXL8*, respectively. In clade III, poplar, with four members, was the most heavily represented of the four species, whereas *Arabidopsis* had only one member. Similar to clade I, the two proteins from poplar in clade IV were most closely related to *SMXLs* from apple. Noteworthily, *PtSMXLs* within a given clade were in a sister relationship to each other, and their genes were close homologs. These results suggest that *SMXL* genes underwent duplication in poplar.

### Chromosomal location, genome synteny, and gene duplication of *PtSMXLs*

**Fig. 2:**
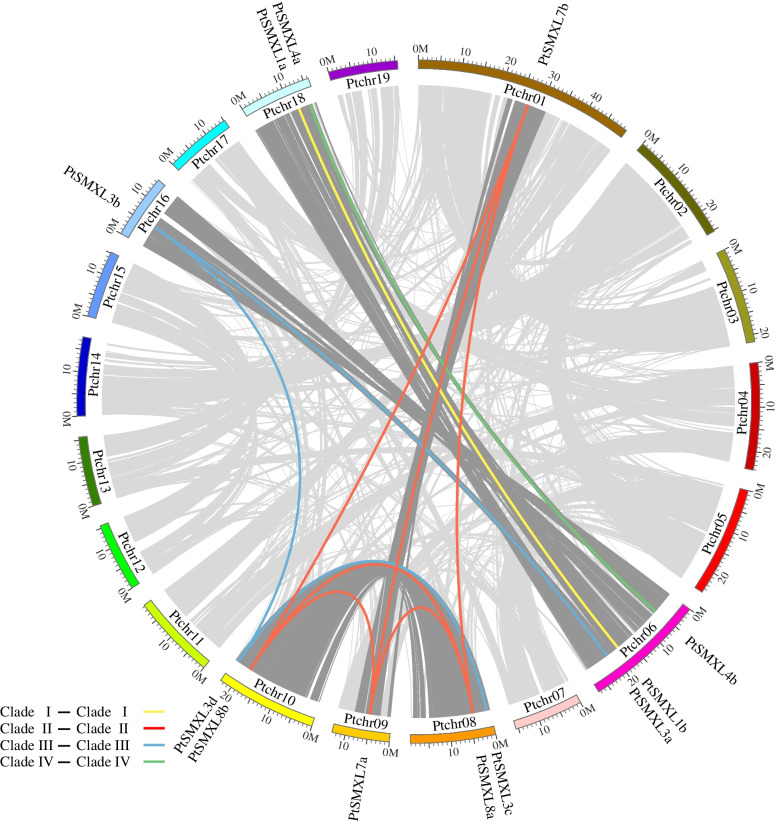
Chromosome distribution and duplication event analysis of 12 *PtSMXL* genes. Ptchr01-Ptchr19 represents 19 poplar chromosomes. Duplication segments identified by MCsanX were connected by grey ribbons, and the duplication segments between chromosomes where 12 *PtSMXL* genes is located were connected by dark grey ribbons. 12 *PtSMXL* genes were labeled at corresponding position of chromosomes, and different colors are used to connect collinearity gene pairs according to different clades. Positions are in Mb

The 12 *PtSMXL* genes identified in this study were unevenly distributed on seven of the chromosomes of poplar (Fig. 2). The largest number of *PtSMXL* genes, three, were distributed on chromosome 6. Two genes each were located on chromosomes 8, 10, and 18, and only one gene was present on each of chromosome 1, 9, and 16. To understand the duplication and evolution of *PtSMXL* genes, we performed a collinearity analysis of the poplar genome. Eleven pairs of *PtSMXL* genes were discovered in duplicated chromosome segments, whereas no gene was identified in tandem repeats, thus suggesting that *PtSMXL* genes mainly expanded via segmental duplication (Table S1). Homologous chromosome segments originating from the salicoid duplication event have been identified in the *P. trichocarpa* genome [24, 27, 28]. We found that 6 of the 11 collinear *PtSMXL* gene pairs were located in these segments (Fig. 2). These six gene pairs represented exactly half of the number of *PtSMXL* genes. We therefore speculated that doubling of the *PtSMXL* gene family took place during the salicoid duplication event and that all duplicated genes were retained during subsequent genome reorganization. To test this hypothesis, we calculated *Ks* values and divergence times of all 11 gene pairs. *Ks* values of the six pairs of *PtSMXL* genes thought to have been doubled during the salicoid duplication event ranged from 0.201 to 0.292, which were far less than the *Ks* values of the other five gene pairs (1.305-1.639) (Table S2). Divergence times of the six gene pairs were approximately 50 Mya (very close to 60-65 Mya). These results support the hypothesis that an ancestor of Salicaceae originally harbored six members of the *SMXL* gene family and that these genes were duplicated in the salicoid event and then retained to form the present 12 members. We also calculated *Ka/Ks* ratios of the six gene pairs, which indicated that these genes all experienced purifying selection after doubling (Table S2).

To compare the evolution and divergence of *SMXLs* among different species, we searched for *SMXL* genes in grape following the same methods used for poplar and uncovered six *SMXL* family members. We then performed two separate collinearity analyses. The first analysis was between poplar and grape; the second one was between poplar and *Arabidopsis*, which possesses eight *SMXL* members. Although 13 *AtSMXL-PtSMXL* gene pairs were identified, their *Ks* values were all greater than 1.874, which suggests that their divergence preceded the salicoid duplication event (Fig. S1, Tables S3 and S4). In contrast, the average *Ks* value of the 12 identified *VvSMXL-PtSMXL* gene pairs was 1.091, which is less than that between poplar and *Arabidopsis* (Fig. S1, Tables S3 and S4). This result indicates that the divergence of *SMXL* genes in poplar from those of grape occurred later than the differentiation from *SMXL* genes of *Arabidopsis*. Moreover, one *VvSMXL* gene corresponded to two *PtSMXL* genes on average according to the collinearity analysis. One possible explanation for this phenomenon is that the grape genome has not undergone recent genome duplication, whereas the number of poplar *SMXL* genes has doubled.

### Gene structure, conserved motif and protein structure characteristics

**Fig. 3:**
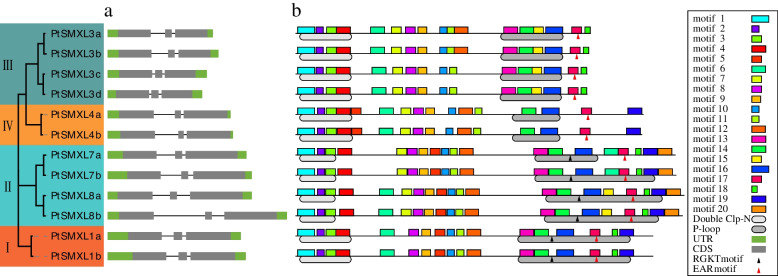
Phylogenetic relationships, gene structure, and motif composition of *PtSMXLs*. **a** The exon-intron distribution of 12 *PtSMXLs*. The exons, introns, and untranslated regions (UTRs) were indicated with different colors. **b** Conservative motif distribution of 12 *PtSMXLs*. These motifs were identified by MEME search

As shown in Fig. 3a, all 12 *PtSMXL* genes were highly conserved in regard to their exon-intron distribution, with the longer exons, exons 1 and 3, situated at either end, and the shorter one, exon 2, separated by two introns. In addition, gene in the same clade, except for clade II, had introns of the same length. In clade II, *PtSMXL8b* had a lengthened intron compared with other members of the clade.

We also identified 20 motifs in the 12 PtSMXL protein sequences via a MEME search and superimposed them on a phylogenetic tree of PtSMXL protein sequences (Table S5, Fig. 3b). According to the results, the N-terminal region of all PtSMXLs, consisting of motifs 1-4 representing the double Clp-N domain, was very conserved (Fig. 3b). Motifs 13-20 at the PtSMXL C-terminal region corresponded to the P-loop NTPase domain, but not all PtSMXLs had all eight motifs (i.e.,13-20). PtSMXL8a/8b in clade II contained all eight motifs, and PtSMXL7a/7b only lacked motif 15. Motifs 15 and 20 were absent from clade I, whereas members of clade III lacked motifs 19 and 20. Clade IV members had the least conserved P-loop NTPase domain, which lacked motifs 13, 15, 18, and 20. Two functional sites of PtSMXLs, the EAR motif and the RGKT motif, were not uncovered by MEME searching, as they possessed a small number of amino acid residues, and were therefore manually identified. All EAR motifs were located in motif 13, and all RGKT motifs were found between motifs 14 and 16. The RGKT motif was present in both clade I and clade II; however, two conserved residues of the RGKT motif in PtSMXL7a and 7b of clade II were replaced with ST or SM (Fig. S2).

**Fig. 4:**
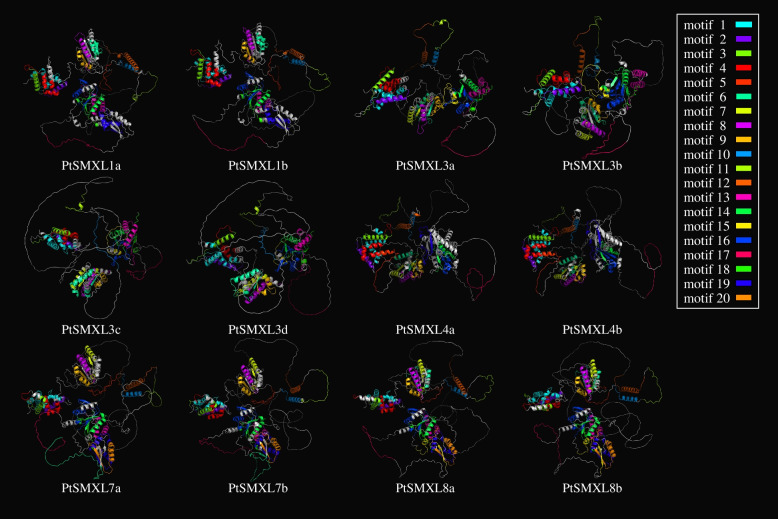
Ribbon diagrams of the predicted three-dimensional structure of 12 *PtSMXLs*. The distribution of conservative motifs is marked with different colors in each structural. The color used by motifs is the same as that in Fig. 3

To further analyze protein structures of *PtSMXL* genes, we downloaded the latest 3D structures of PtSMXL proteins predicted by AlphaFold from the AlphaFold protein structure database and labeled their 20 predicted motifs with the same color scheme as in (Figs. 3 and 4). Consistent with previous studies, SMXL proteins could be spatially divided into four domains Double Clp-N, D1, middle region, and P-loop NTPase, these four domains were separated by intrinsically disordered regions (IDRs) [7, 17]. The identified conserved motifs were mainly located within the four domains, not in the IDRs; however, motif 17 and the EAR motif within it were located in an IDR.

### *Cis*-acting elements of *PtSMXL* genes

**Fig. 5:**
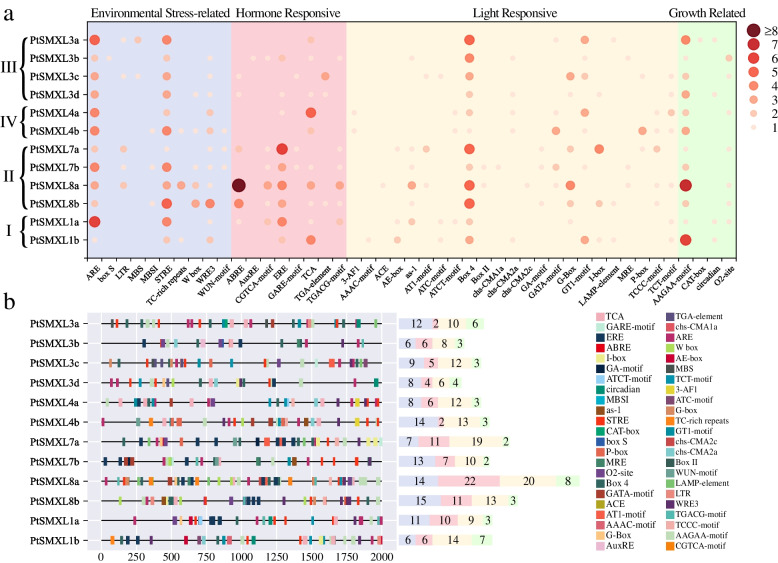
Analysis of *Cis*-acting element of 12 *PtSMXL* promoters. **a** Enrichment of each *Cis*-acting element. The size and color of the dots represent the number of *Cis*-acting elements. **b** Distribution of *Cis*-acting elements in *PtSMXL* promoter region, and the proportion of four classes of elements

*Cis*-acting elements play an important role in regulation of gene expression [29]. To analyze the expression regulation and response mechanism of *PtSMXL* genes, we retrieved the 2000-bp sequences upstream from the translation start codons of *PtSMXL* genes and identified putative *Cis*-acting elements using the PlantCARE database. A series of *Cis*-acting elements were identified in the 12 *PtSMXL* promoters. We selected 408 elements with functional annotation for further analysis. These 408 elements could be roughly divided into four classes: environmental stress-related, hormone-responsive, light-responsive, and plant growth-related elements (Fig. 5a). Light-responsive elements (146/408), followed by environmental stress-related elements (123/408), were the most abundant of the four classes. Furthermore, the proportion of hormone-responsive elements in *PtSMXL* clades I and II (27.5%) was much higher than that in clades III and IV (15.1%) (Fig. 5b). Four elements, namely, ARE, STRE, Box 4, and AAGAA-motif, were present in all 12 *PtSMXLs* and accounted for 36.5% of the total four classes elements. ARE and STRE belong to the class of environmental stress-related elements, whereas Box 4 and AAGAA-motif are light-responsive and plant growth-related elements, respectively. The promoter of *PtSMXL8a* had the largest number of these four classes of elements, 1.5–2.9 times more than other genes, and also had the highest proportion of hormone-responsive elements. These results indicate that the 12 *PtSMXLs* may be involved in diverse physiological activities.

### Expression analysis of *PtSMXL* genes

The expression patterns of *PtSMXLs* in different tissues were measured by qRT-PCR, which revealed that expression levels differed significantly among tissues and genes (Fig. S3). Most *PtSMXL* genes were highly expressed in dormant axillary buds or mature stems. For example, *PtSMXL1a*, *PtSMXL3d*, *PtSMXL4b*, and *PtSMXL7b* were highly expressed in dormant axillary buds, whereas *PtSMXL1b*, *PtSMXL3a*, and *PtSMXL3b* were highly expressed in mature stems. Except for *PtSMXL7a*, members of clades I and II were weakly expressed in roots. In addition, all members of clade III had high expression levels in stems. Among these genes, *PtSMXL3a* and *PtSMXL3b* had high expressions in mature stems, whereas *PtSMXL3c* and *PtSMXL3d* were strongly expressed in young stems. Noteworthily, the expression of *PtSMXL8a* varied by more than 20-fold across different tissues. The contrasting expression patterns of *PtSMXLs* in various tissues suggest that the members of *PtSMXLs* may function in different tissues.

**Fig. 6:**
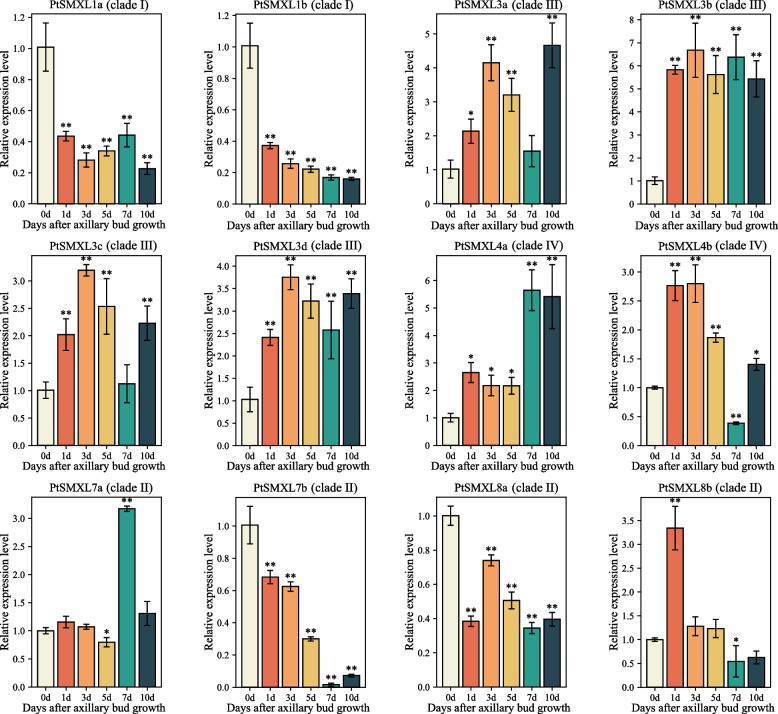
Expression patterns of *PtSMXL* genes at different axillary bud growth stages. Complete axillary buds were used as the material. Sampling occurred 0, 1, 3, 5, 7, and 10 days after axillary bud growth, and the relative expression level of the sample at 0 days was set to 1. Asterisks indicate statistically significant differences between 0-day control group and other groups (* *P* ≤ 0.05, ** *P* ≤ 0.01)

**Fig. 7:**
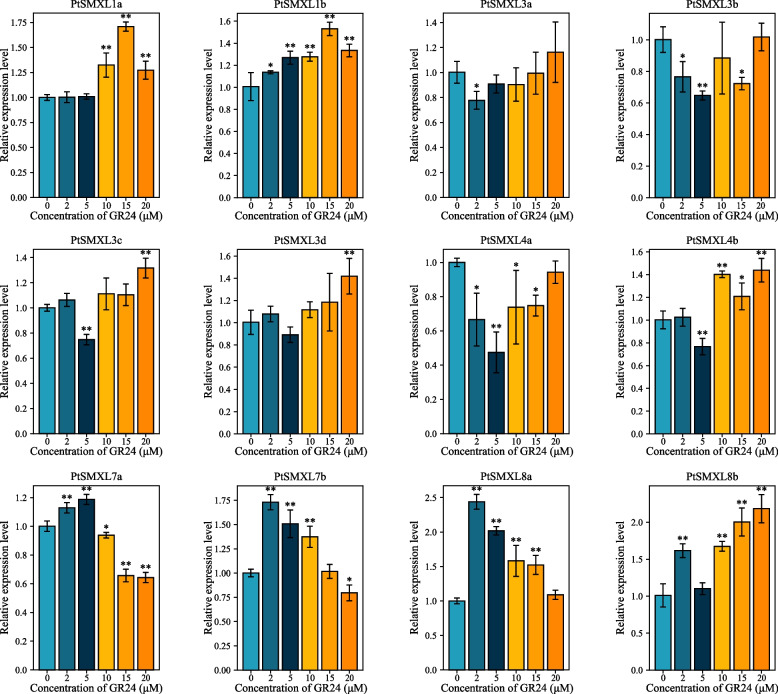
Expression patterns of *PtSMXL* gene under treatment with different GR24 concentrations. Whole seedling stems were used as the experimental material. Sampling at 30 days after processing with 0, 2, 5, 10, 15, and 20 μM GR24, and the relative expression level of the sample treated with 0 μM GR24 was set to 1. Asterisks indicate statistically significant differences between 0 μM control group and other groups (* P ≤ 0.05, ** P ≤ 0.01)

To further study the relationship between *PtSMXLs* and axillary bud branching, we analyzed *PtSMXL* expressions at different stages after axillary bud growth begins. The observed expression patterns of *PtSMXLs* at different axillary bud growth stages could be roughly divided into two categories (Fig. 6). In particular, members of clades III and IV had similar expression patterns: weak expression during the initial stage of axillary bud growth and a subsequent increase. In contrast, members of clades I and II, except for *PtSMXL7a*, were highly expressed at the initial stage of axillary bud growth and then expressed weakly thereafter. The expression of *PtSMXL7a* did not change significantly during the first 5 days of axillary bud growth, but this gene was significantly upregulated 7 days after axillary bud growth begins. After axillary bud growth begins, the expression of *PtSMXL7b* exhibited an obvious downward trend. Seven days after axillary bud growth begins, the expression of *PtSMXL7b* had declined significantly and was only approximately one-sixtieth of its initial level. We also investigated the response of *PtSMXLs* to treatment with rac-GR24, a synthetic SL analog (Fig. 7). When the concentration of added GR24 was at or below 5 μM, five genes were significantly upregulated and five were significantly downregulated. At a GR24 concentration of 10 μM or higher, however, seven and three genes were significantly upregulated and downregulated, respectively. Among these genes, the expressions of two, *PtSMXL7b* and *PtSMXL8a*, followed similar trends with increasing GR24 concentrations. When the GR24 concentration was low (2 μM), their expression levels significantly increased, but as the GR24 concentration continued to rise, the levels of their expression show a gradually decreasing trend. At a GR24 concentration of 2 μM, the expression of *PtSMXL8a* was 2.5-fold higher than that of the control, which is consistent with the observation that *PtSMXL8a* had more hormone-responsive elements according to our *Cis*-element analysis.

### Interaction of clade II PtSMXLs with PtD14a/b

**Fig. 8:**
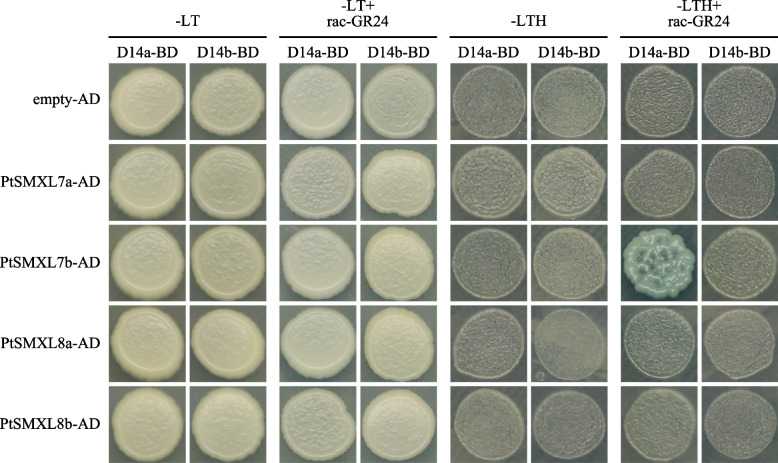
Interaction analysis of PtSMXL clade II members with PtD14a/b by yeast two hybrid experiments. D14a and D14b were fused to GAL4-BD. SMXL7a, SMXL7b, SMXL8a and SMXL8b were fused to GAL4-AD. Yeast cells were co-transformed with D14a/D14b and SMXL7a, SMXL7b, SMXL8a, SMXL8b or empty AD vector. Yeast cultures were spotted onto selective growth medium -LT or -LTH (−H, −His; −L, −Leu; −T, −Trp). These media were supplemented with 20 μM GR24, or 0.02% acetone control. Images show growth after 3 d at 30 °C

Members of *PtSMXL* clade II share a close homologue relationship with rice *OsD53* and *Arabidopsis AtSMXL7*. Proteins encoded by these two genes can interact with D14 in an SL-dependent manner to participate in the SL signaling pathway [5, 12]. Therefore, we reasoned that members of SMXL clade II in poplar may also can interact with PtD14a/b. Yeast two-hybrid experiments were employed to verify this hypothesis. The results showed that all yeast strains were unable to grow on selective medium without the addition of GR24 (SD/−LTH). However, only the strains containing PtSMXL7b and D14a was able to grow and turn X-α-GAL blue on selective medium supplemented with 20 μM GR24 (SD/−LTH + GR24) (Fig. 8). This suggests a GR24 dependent interaction between PtSMXL7b and PtD14a in yeast cell. Therefore, we further believe that *PtSMXL7b* may play an important role in the branching development of poplar.

### Overexpression of *PtSMXL7b* improves axillary bud growth

**Fig. 9:**
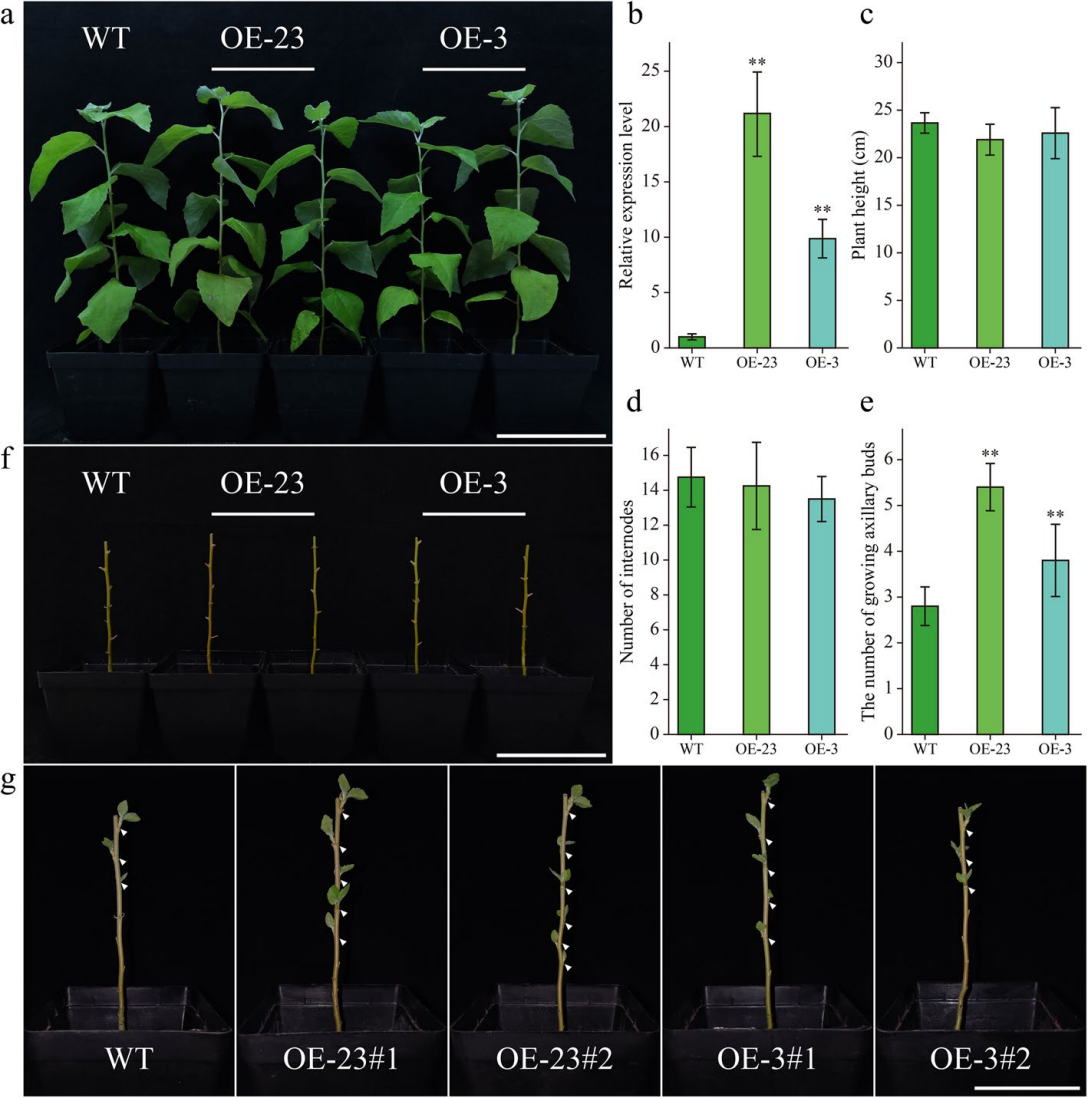
The overexpression of *PtSMXL7b* promotes the growth of poplar axillary buds. **a** Phenotypic identification of poplar. **b** Relative expression level of *PtSMXL7b* gene in overexpression and wild-type lines. **c** Plant height of overexpression and wild-type lines. **d** Number of internodes in overexpression and wild-type lines. **e** The number of axillary buds that grew in the overexpression and wild-type lines after removing the first five internodes for 10 days. **f** Poplars in which the first five internodes were removed (**g**) Phenotypic identification of poplar after removing the first five internodes for 10 days. The grown axillary buds are pointed out with white triangles. Data are means ± SE (*n* = 10), and were analyzed by Student’s t tests (*p < 0.05 and **p < 0.01). Scale bars, 10 cm (**a**), 5 cm (**f**). The “#” represent the duplication in the line

According to our expression quantity experimental results, the expression level of *PtSMXL7b* gene was significantly decreased during axillary bud growth, and low concentrations of GR24 were able to induce an increase in its expression levels (Figs. 6 and 7). Additionally, we observed the interaction between PtSMXL7b and PtD14a in the yeast two-hybrid experiment. Therefore, we believe that *PtSMXL7b* plays a role in the growth of poplar axillary buds. In order to determine the function of *PtSMXL7b*, we created the overexpression poplar of *PtSMXL7b* (Fig. 9a). Two overexpression lines OE-3 and OE-23 were selected for phenotypic testing, and *PtSMXL7b* of these two lines were overexpressed 9 and 21 times, respectively (Fig. 9b). After 2 months of growth under the same environmental conditions, there was no significant difference in plant height or internode number between overexpression lines and WT (Fig. 9c, d). To further investigate the effect of overexpressing *PtSMXL7b* on axillary bud growth, we removed the apical buds of all lines. When the apical buds were removed for 10 days, axillary bud growth was observed on all lines (Fig. 9f, g). However, the number of axillary buds growing in both OE-3 and OE-23 lines was significantly higher than WT (Fig.9e). These results suggest that *PtSMXL7b* is able to positively regulate the growth of axillary bud in poplar, and thereby promote poplar branching.

## Discussion

Branch development is an important aspect of tree crown shape formation-a process that affects the stand density and light energy utilization rate of trees and thus is important for afforestation [30, 31]. Plant branch development is regulated by many plant hormones, including SLs [32]. Recent studies have shown that SLs play an important role in regulating plant shoot branching [3]. SL signaling is controlled by a variety of key proteins [5], including SMXLs that function as key repressors in SL signaling [4]. The functions of SMXLs have been widely studied in the model plant *Arabidopsis* and the crop plant rice, but little is known about their characteristics in the forest tree poplar. In this study, we identified 12 *P. trichocarpa SMXL* genes, which is larger than the number previously found in *Arabidopsis* (8) [22] and apple (10) [23]. Past evolutionary studies have shown that *SMXL* genes of angiosperms can be divided into four major clades [6]. In poplar, the number of genes in clades I and IV is consistent with that of *Arabidopsis*, whereas the number in clades II and III is higher in the former species than in the latter. The expanded size of clades II and III in poplar may explain the difference in branch development between poplar and *Arabidopsis*. Because a recent genome doubling event unique to Salicaceae has been inferred in previous studies [24], we speculated that the *PtSMXL* family has been affected by this duplication. We therefore performed a genomic collinearity analysis of *PtSMXLs*, which indicated that the number of *SMXL* genes of poplar may have doubled after the salicoid genome duplication event—expanding from possibly six members in the ancestor of Salicaceae to the present 12 members. Interestingly, and in accordance with this result, poplar has two ancestral genes, *SMXL7* and *SMXL8*, in *SMXL* clade II, which supports the past view that eudicots further diverged into two separate subgroups, consequently separating *SMXL6*/7 and *SMXL8* in clade II [6].

The *SMXL* gene family is widely present in land plants but has a highly conserved structure [6, 9]. *PtSMXL* genes consist of three exons separated by two introns. Exon 2 is very short and is located in the middle of two long exons. These characteristics are consistent with the situation in apple, which indicates that *SMXL* gene structure is quite conserved among different species [23]. In addition, we found that the double Clp-N domain in the protein N terminal is very conserved among PtSMXLs, whereas the P-loop NTPase domain in the C terminal varies greatly among clades. According to studies of various species, the double Clp-N domain is highly conserved among SMXLs and is likely related to the nuclear localization of these proteins [8]. In *Arabidopsis*, the P-loop NTPase domain is related to SL-dependent ubiquitination degradation of SMXLs [7, 17]. More importantly, the key RGKT motif is located in the P-loop NTPase domain [6]. Mutations in the RGKT motif led to an increase in the number of branches (tillering) in both *Arabidopsis* and rice [5, 8]. Interestingly, we found that PtSMXL7a/b have an atypical RGKT motif whose amino acid residue GK has been replaced in the two proteins by ST and MT, respectively. This atypical RGKT motif may affect the function of PtSMXL7a/b in SL signaling and could reflect the differentiation of *SMXLs* in poplar, but future experiments are needed to examine this hypothesis in detail.

The expression pattern of a gene provides important clues to its function. Previous studies have shown that different clades of *SMXL* genes in apple have different tissue-specific expression patterns [23]. Similarly, we found that *PtSMXLs* in different tissues of poplar have significantly different expression levels. In various species, *SMXLs* in clade II are very weakly expressed in roots [4, 33]. In our study, members of *PtSMXL* clade II, with the exception of *PtSMXL7a*, had a similar expression pattern. In addition, most genes were highly expressed in dormant axillary buds (four) and mature stems (three), which suggests that these genes are involved in axillary bud and stem development. Members of *SMXL* clade II mainly function in SL signaling as signal inhibitors and thereby regulate several physiological processes, including inhibition of shoot branching [4, 6]. In our study, the expressions of *PtSMXL7b* and *PtSMXL8a* from *PtSMXL* clade II were significantly downregulated after axillary bud growth begins and continued to decrease. This trend suggests that *PtSMXL7b* and *PtSMXL8a* participate in the onset of axillary bud growth in a negative way. Previous research has shown that *SMXLs* can negatively regulate their own expressions, and *SMXL* proteins treated with GR24 are degraded in a GR24-dependent manner, de-repressing their own transcriptional repression, resulting in increased transcript levels [16, 22, 34]. To study the response of *PtSMXLs* to SLs, we measured *PtSMXL* expression levels in stems treated with different concentrations of GR24. When the concentration of GR24 was low (no more than 5 μM), all clade-II *PtSMXLs* were significantly upregulated. As the concentration of GR24 was increased, *PtSMXL7b* and *PtSMXL8a* expressions followed the same downward trend. These results are consistent with previous reports that SLs can induce the upregulation of *SMXL* gene expression in many species [4, 22, 33].

In rice, the SMXL homolog D53 is a target of SL-induced ubiquitination degradation taking place through its interaction with the SL receptor D14 [5]. SMXL6/7/8, which are orthologs of D53, are thought to play the same role in *Arabidopsis* [12]. OsD53 and AtSMXL6/7/8 all belong to clade II of the *SMXL* family [6]. In previous studies, the interaction of OsD14 with OsD53 was verified by yeast-two hybrid assays [5]. A different result was obtained in *Arabidopsis*, however, as the interaction of AtSMXL6/7 with AtD14 was confirmed, but AtSMXL8 did not interact with AtD14 [12]. Here, four members of SMXL clade II are present in poplar: PtSMXL7a/7b, homologous to AtSMXL6/7, and PtSMXL8a/8b, homologous to AtSMXL8. We performed yeast two-hybrid experiments to determine the interaction relationships of these four members with PtD14a/b. However, the results showed that only PtSMXL7b exhibited a GR24 dependent interaction with PtD14a. These differing results may be a consequence of the differentiation of clade II in eudicots [6]. But it is very important to verify the results of yeast two-hybrid assay in vivo. Whether other members of clade II can interact with PtD14a/b in vivo remains to be verified by subsequent experiments.

SLs are able to inhibit plant branching by inducing the ubiquitination degradation of *D53-like/SMXLs* [4, 5, 7, 8]. Past studies have shown that overexpression of *AtSMXL6* in *Arabidopsis* can promote branching [12]. Here we demonstrated that *PtSMXL7b* had a positive effect on axillary bud outgrowth in poplar by constructing poplars overexpressing *PtSMXL7b*. This is consistent with previous research results [4, 12]. In the case where there was no significant difference in plant height and internode number, the overexpression lines have grown more axillary buds than WT. This indicates that SLs can affect plant branching by influencing axillary bud growth. In past studies, mutants with impaired signal transduction of SLs mostly presented with the dwarf phenotype [5, 8, 11]. However, in our study, simple overexpression of *PtSMXL7b* did not show significant plant height variation. This may be due to the fact that overexpression at the transcriptional level is not sufficient to override its degradation at the protein level or that there is functional redundancy between *PtSMXL7b* and other members of the *SMXL* family in poplar [5, 6, 8]. In conclusion, *PtSMXL7b* positively regulates branching development in poplar, but the specific mechanism needs further investigation.

## Conclusions

In this study, 12 *PtSMXL* genes were identified in poplar genome, and phylogenetically divided into four clades. A genomic collinearity analysis provided evidence that *PtSMXLs* have undergone doubling and purification selection. According to the protein structure and motif analysis, all PtSMXLs are structurally very conserved, but PtSMXL7a and PtSMXL7b have atypical RGKT motif. We also analyzed *PtSMXL Cis*-acting element, investigated *PtSMXLs* expression pattern in different tissue, and monitored their expressions after axillary bud growth begins and in response to GR24. In addition, a yeast two-hybrid assay verified that PtSMXL7b in PtSMXL clade II can interacts with PtD14a in a GR24 dependent manner. Finally, we proved that *PtSMXL7b* can promote the growth of axillary buds by constructing poplar overexpressing *PtSMXL7b*.

## Methods

### Plant materials

Roots, young leaves (first to third leaves of shoots), mature leaves (4th to 7th leaves of shoots), young stems (first to third internodes of shoots), mature stems (4th to 7th internodes of shoots), and dormant axillary buds were sampled from 5-year-old individuals of hybrid 84 K (*Populus alba × P. glandulosa*) grown under natural conditions in an experimental plot at Shandong Agricultural University (36°10′ N, 117°9′ W). These materials are used for quantitative PCR experiments measuring the expression levels of genes in different tissues.

The shoots of current year from 5-year-old poplar trees were collected in November. The upper axillary buds were dormant at this time. These shoots were cultured in water under same environmental conditions (24 °C and 16 h light / 8 h dark). For the growth of dormant axillary buds, the first three internodes of all shoots were removed. After 0, 1, 3, 5, 7, and 10 days of culture, the axillary buds were collected. These materials are used for quantitative PCR experiments measuring the expression levels of genes in axillary buds during different stages of axillary bud growth.

Poplar seedlings in tissue culture were inoculated in half-strength MS medium supplemented with 0, 2, 5, 10, 15, or 20 μM of rac-GR24, a synthetic strigolactone analog. After 30 days of culture, the intact stems were collected for use in quantitative PCR experiments to measure the effects of different concentrations of GR24 on gene expression.

Each treatment was repeated three times. All samples were quickly frozen in liquid nitrogen and then stored at − 80 °C for later RNA extraction.

### Identification of the SMXL gene family in *P. trichocarpa*

The latest *P. trichocarpa* genome (v4.1) and corresponding protein sequences were downloaded from Phytozome v13.0 (https://phytozome-next.jgi.doe.gov/). A hidden Markov model (HMM) search was used to identify *SMXL* genes in annotated protein databases derived from the *P. trichocarpa* genome. The HMM profile was constructed from a multiple sequence alignment of published *Arabidopsis* SMXL protein sequences [12, 35], and the hmmersearch was then performed with an E-value cutoff of 1 × 10^− 20^. Hmmersearch was executed through the graphical interface tool SPDE v2.0 [36]. As a result, a total of 21 proteins were searched. After that, we used the SUPERFAMILY (https://supfam.org/) and the NCBI CDD (https://www.ncbi.nlm.nih.gov/Structure/cdd/wrpsb.cgi) databases to verify the presence of conserved double Clp-N and P-loop ATPase domains in these proteins, and the EAR motif ([FL]-D-L-N) was confirmed by manual searching, the proteins that did not contain conserved domains and motif were removed. After removal of redundant transcripts sequences, remaining 12 sequences were subjected to subsequent analyses. In addition, physical properties, including protein length, molecular weight (MW), theoretical isoelectric point (pI), instability index (II) and grand average of hydropathicity (GRAVY) were calculated by ExPasy server (https://web.expasy.org/protparam/).

### Phylogenetic and genetic structure analysis

To align several important motifs of SMXLs, the SMXL protein sequences of *Arabidopsis* [12], rice [33], and apple [23], were downloaded from NCBI database (https://www.ncbi.nlm.nih.gov/) and aligned with those of *P. trichocarpa* using the E-INS-I method in MAFFT v7.49 with default parameters [37]. A maximum-likelihood phylogenetic analysis was performed under the Jones-Taylor-Thornton amino acid substitution model in MEGA 6.0 with 1000 bootstrap replicates, and the resulting tree was visualized with Evolview (http://www.evolgenius.info/evolview/). Referring to *Arabidopsis SMXL* genes, the *SMXL* genes of *P. trichocarpa* were renamed according to their positions in the phylogenetic tree. In addition, information on the distribution of *PtSMXL* gene introns and exons was extracted using SPDE2.0 software and visualized with Evolview.

### Analysis of motifs and promoter *Cis*-elements

Conserved motifs of PtSMXL protein were identified using the MEME tool (https://meme-suite.org/tools/meme) with default parameters except for the following settings: classic mode; zero or one occurrence per sequence; and number of motifs = 20. Several important, previously reported motifs, including RGKT and EAR motifs, that were not identified by MEME were manually located and recorded [17]. The 3D structures of PtSMXLs were downloaded from AlphaFold protein structure database (https://alphafold.com/), and motifs were labeled with different colors in PyMOL (http://www.pymol.org/pymol).

The 2000-bp upstream sequence regions of all *PtSMXL* genes were extracted with SPDE2.0 software, and analyzed using the PlantCARE tools (http://bioinformatics.psb.ugent.be/webtools/plantcare/html/).

### Chromosomal distribution, genome synteny, and Ka/Ks ratios

Information on chromosomal positions of *PtSMXLs* was obtained from the genome annotation file. Genome collinearity was analyzed in MCScanX and collinear pair of *SMXLs* were identified using the downstream analysis function of MCScanX [38]. Synonymous (*Ks*) and non-synonymous (*Ka*) substitution rates and *Ka/Ks* ratios of duplicated *SMXL* gene pairs were calculated with TBtools [39]. Divergence time was calculated according to the following formula: T = *Ks* / (2 × 2.5 × 10-9) × 10-6 million years ago (Mya) [24, 40].

### Expression analysis

*PtSMXL* gene expression profiles were analyzed in roots, young leaves, mature leaves, young stems, mature stems, and dormant axillary buds of 84 K poplar. In addition, the response of *PtSMXL* genes to GR24 was studied in seedlings treated with six concentrations (0, 2, 5, 10, 15, or 20 μM) of GR24, and *PtSMXL* expression profiles during axillary bud growth were examined in axillary buds at six different stages after the begins of axillary bud growth (0, 1, 3, 5, 7, and 10 days). The more detailed description of materials is presented in the Plant materials section. Total RNA was isolated from each sample using a SteadyPure Plant RNA Extraction Kit AG21019 (Accurate Biotechnology [Hunan] Co., Ltd) and then quality-checked on a NanoDrop One UV spectrophotometer (Thermo Scientific, USA). First-strand cDNA synthesis was carried out with DNA-free RNA using Evo M-MLV Plus 1st Strand cDNA Synthesis Kit AG11615 (AG). Real-time quantitative PCR (qRT-PCR) was performed using a SYBR Green Premix Pro Taq HS qPCR Kit AG11701 (AG) on a CFX-96 real-time PCR detection system (Bio-Rad, USA). Each experiment consisted of three independent biological replicates, with three technical replicates per sample. The β-actin gene was used as an internal control. Relative expression levels of each target gene were analyzed the 2^-∆∆CT^ method [41]. Sequences of all primers used are listed in Table S6.

### Yeast two-hybrid assays

The CDSs of *PtSMXL7a*, *PtSMXL7b*, *PtSMXL8a*, *PtSMXL8b*, and *PtD14a* and *PtD14b* were amplified by PCR. *PtSMXL7a/7b/8a/8b* were then cloned into the pGADT7 vector, *PtD14a/b* were cloned into the pGBKT7 vector [42]. The resulting constructs were co-transformed into yeast strain Y2H, and growth on SD/−Leu/−Trp plates was used to confirm the presence of target transgenes. To assay protein interactions, transformed yeast were tested on SD/−His/−Leu/−Trp + X-α-gal (4 mg/ml-1) with or without GR24 (20 μM). The cultures were incubated at 30 °C and observed after 3 days.

### Plant transformation

To generate the transgenic poplar, the *PtSMXL7b* was inserted into the expression vector PBI121 between *BamH* I and *Sac* I restriction sites by homogenous recombination method, was made to be driven by the 35S promoter. Then, the overexpression vector was transformed into 84 k poplar via Agrobacterium-mediated transformation according to a previously reported method [43]. To identify transgenic plants, genomic DNA of different lines was extracted using the CTAB method, and gene-specific primers were used to amplify the 795-bp Neomycin Phosphotransferase II (NPTII) gene fragment (Table S6). In addition, the expression level of the *PtSMXL7b* in the transgenic plants were determined by using qRT-PCR (Table S6). All plants were grown under a light and dark cycle of 16 h and 8 h at 24 °C.

### Phenotype test of transgenic poplar

Two-month-old transgenic poplar were selected for phenotypic testing. First, the internode where the first fully expanded lead resided was defined as the first internode. Plant height and internode number were measured for the three lines, and at least ten replicates were measured for each line. Subsequently, the first 5 internodes of all plants were removed and cultured for 10 days to promote axillary bud growth. After 10 days, count the number of axillary buds grown in each line, and count at least 10 replicates.

## Supplementary Information

**Additional file 1: Supplementary Fig. S1.** Genomic collinearity analysis of interspecies between poplar and grape, and between poplar and Arabidopsis. Gray lines represent the collinear blocks between two plants, gene pairs from different evolutionary branches are connected in different colors. **Supplementary Fig. S2.** Conservative RGKT motif and EAR motif of SMXL from different species. The conserved RGKT motif and EAR motif in SMXL proteins of poplar, Arabidopsis, and rice. Conserved amino acids in both motifs are marked by red boxes. **Supplementary Fig. S3.** Expression patterns of PtSMXLs gene in different tissues. Take roots (R), young stems (Ys), mature stems (Ms), young leaves (Yl), mature leaves (Ml), and dormant axillary buds (Bd) as samples, and the relative expression level in root was set to 1. Asterisks indicate statistically significant differences between roots and other tissues (* *P* ≤ 0.05, ** *P* ≤ 0.01).

**Additional file 2: Supplementary Table S1.** The duplication relationships of *PtSMXLs*. The two genes in each row in the table represent the collinear gene pairs identified by MCScanX. The GFF file provides information on the location of genes on chromosomes. The genes marked in red are located in chromosomal segments that are duplicated during salicoid duplication event.

**Additional file 3: Supplementary Table S2.** The *Ks* and *Ka* values of duplicated *PtSMXLs* gene pairs. The gene pairs marked in red are those that have been duplicated during salicoid duplication event.

**Additional file 4: Supplementary Table S3.** The duplication relationships between *PtSMXLs* and *AtSMXLs*, and between *PtSMXLs* and *VvSMXLs*. The genes of poplar are represented by gene names, and the genes of *Arabidopsis* and grape are represented by genome sequence numbers. The lines indicate that the two genes do not have collinearity.

**Additional file 5: Supplementary Table S4.** The *Ks* and *Ka* values of duplicated gene pairs of interspecies genomic collinearity.

**Additional file 6: Supplementary Table S5.** Conserved motif sequence of PtSMXLs.

**Additional file 7: Supplementary Table S6.** The primer designed for qRT-PCR and vector construction.

## Data Availability

The reference genome assembly used for data analysis was obtained from Joint Genome Institute (JGI) Phytozome. All data generated or analyzed during this study are included in this article (and its supplementary information files) or are available from the corresponding author on reasonable request.

## Abbreviations

SL: Strigolactone
SMXL: SUPPRESSOR of MAX2 1-LIKE
EAR: ETHYLENE-RESPONSE FACTOR Amphiphilic Repression
RGKT: Arg-Gly-Lys-Thr
KAR: KARRIKIN
Mya: Million years ago
*Ks*: Synonymous
*Ka*: Non-synonymous
IDRs: Intrinsically disordered regions
WT: Wild type
