# Supplementary material for: Genome-wide identification and analysis of the SUPPRESSOR of MAX2 1-LIKE gene family and its interaction with DWARF14 in poplar
Source: BMC Plant Biol. 2023 Feb 22;23:105. doi: 10.1186/s12870-023-04118-w (PMC9945404; doi:10.1186/s12870-023-04118-w)
Supplement: Supplementary file 1 — Additional file 1: Supplementary Fig. S1. Genomic collinearity analysis of interspecies between poplar and grape, and between poplar and Arabidopsis. Gray lines represent the collinear blocks between two plants, gene pairs from different evolutionary branches are connected in different colors. Supplementary Fig. S2. Conservative RGKT motif and EAR motif of SMXL from different species. The conserved RGKT motif and EAR motif in SMXL proteins of poplar, Arabidopsis, and rice. Conserved amino acids in both motifs are marked by red boxes. Supplementary Fig. S3. Expression patterns of PtSMXLs gene in different tissues. Take roots (R), young stems (Ys), mature stems (Ms), young leaves (Yl), mature leaves (Ml), and dormant axillary buds (Bd) as samples, and the relative expression level in root was set to 1. Asterisks indicate statistically significant differences between roots and other tissues (* P ≤ 0.05, ** P ≤ 0.01). [file 12870_2023_4118_MOESM1_ESM.pdf]

Genome-wide identification and analysis of the SUPPRESSOR of MAX2 1-LIKE gene family and its interaction with DWARF14 in poplar

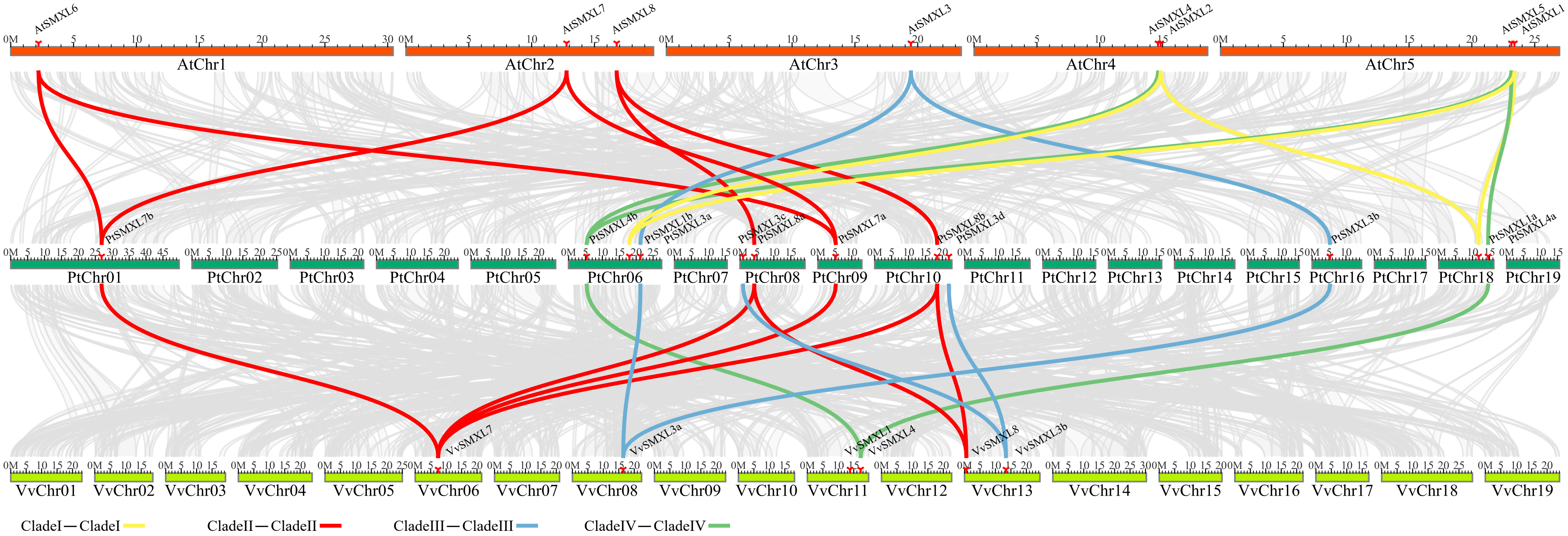

Supplementary Figure S1 Genomic collinearity analysis of interspecies between poplar and grape, and between poplar and Arabidopsis. Gray lines represent the collinear blocks between two plants, gene pairs from different evolutionary branches are connected in different colors.

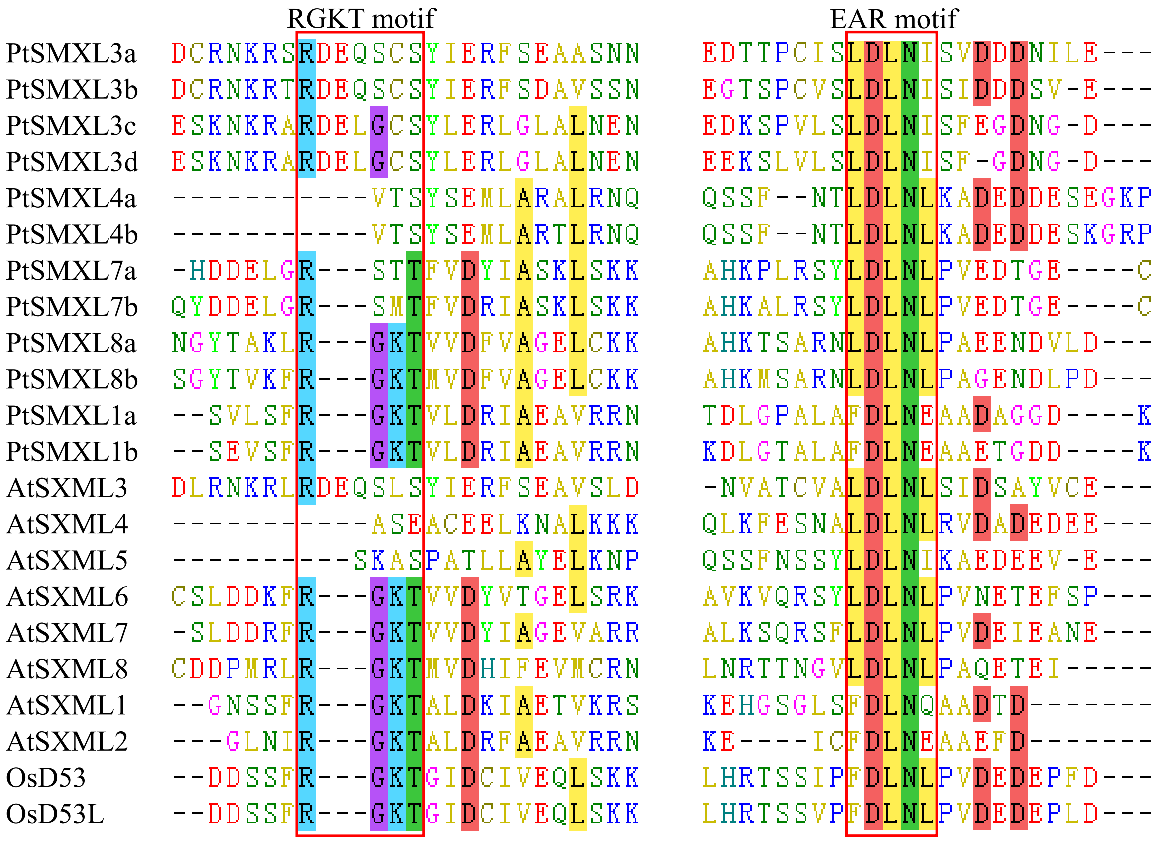

Supplementary Figure S2 Conservative RGKT motif and EAR motif of SMXL from different species. The conserved RGKT motif and EAR motif in SMXL proteins of poplar, Arabidopsis, and rice. Conserved amino acids in both motifs are marked by red boxes.

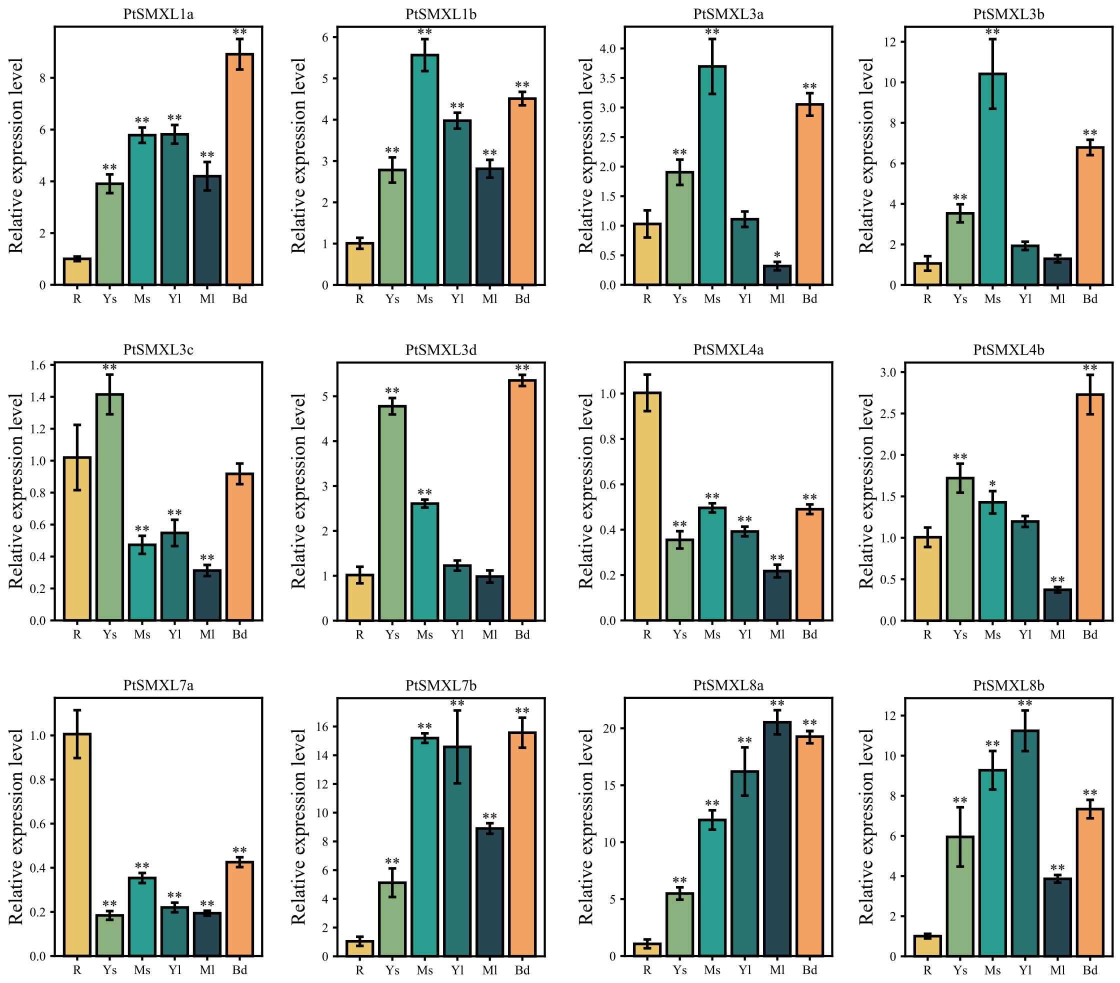

Supplementary Figure S3 Expression patterns of PtSMXLs gene in different tissues. Take roots (R), young stems (Ys), mature stems (Ms), young leaves (Yl), mature leaves (Ml), and dormant axillary buds (Bd) as samples, and the relative expression level in root was set to 1. Asterisks indicate statistically significant differences between roots and other tissues (* $P \leq 0.05$, ** $P \leq 0.01$).
